# 2,2′-[*o*-Phenylenebis(methylenethio)]bis(pyridine *N*-oxide)

**DOI:** 10.1107/S160053680902073X

**Published:** 2009-06-06

**Authors:** Chao-Yan Zhang, Qian Gao, Yue Cui, Ya-Bo Xie

**Affiliations:** a College of Environmental and Energy Engineering, Beijing University of Technology, Beijing 100022, People’s Republic of China

## Abstract

In the title compound, C_18_H_16_N_2_O_2_S_2_, the benzene ring makes dihedral angles of 7.41 and 86.59° with the two outer pyridine N-oxygen rings. Two short intramolecular C—H⋯S contacts occur. The crystal packing is stabilized by C—H⋯O hydrogen bonds, C—H⋯π inter­actions and weak π–π staking inter­actions [centroid–centroid distance 3.7596 (7) Å].

## Related literature

For a related stucture, see: Han *et al.* (2005 ▶). For thio­ether compounds, see: Xie *et al.* (2005 ▶).
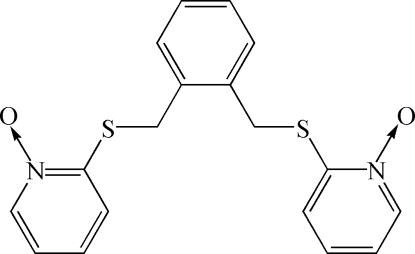


## Experimental

### 

#### Crystal data

C_18_H_16_N_2_O_2_S_2_
*M* *_r_* = 356.47
Monoclinic, 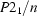

*a* = 7.5075 (15) Å
*b* = 17.810 (4) Å
*c* = 12.480 (3) Å
β = 105.20 (3)°
*V* = 1610.3 (7) Å^3^
*Z* = 4
Mo *K*α radiation
μ = 0.34 mm^−1^
*T* = 293 K
0.34 × 0.28 × 0.16 mm

#### Data collection

Bruker SMART CCD area-detector diffractometer
Absorption correction: none
9681 measured reflections
3782 independent reflections
3145 reflections with *I* > 2σ(*I*)
*R*_int_ = 0.017

#### Refinement

*R*[*F* ^2^ > 2σ(*F* ^2^)] = 0.030
*wR*(*F* ^2^) = 0.083
*S* = 1.02
3782 reflections
225 parameters
H atoms treated by a mixture of independent and constrained refinement
Δρ_max_ = 0.25 e Å^−3^
Δρ_min_ = −0.20 e Å^−3^

### 

Data collection: *SMART* (Bruker, 1998 ▶); cell refinement: *SAINT* (Bruker, 1998 ▶); data reduction: *SAINT*; program(s) used to solve structure: *SHELXS97* (Sheldrick, 2008 ▶); program(s) used to refine structure: *SHELXL97* (Sheldrick, 2008 ▶); molecular graphics: *SHELXTL* (Sheldrick, 2008 ▶); software used to prepare material for publication: *SHELXTL*.

## Supplementary Material

Crystal structure: contains datablocks I, global. DOI: 10.1107/S160053680902073X/at2798sup1.cif

Structure factors: contains datablocks I. DOI: 10.1107/S160053680902073X/at2798Isup2.hkl

Additional supplementary materials:  crystallographic information; 3D view; checkCIF report

## Figures and Tables

**Table 1:** Hydrogen-bond geometry (Å, °)

*D*—H⋯*A*	*D*—H	H⋯*A*	*D*⋯*A*	*D*—H⋯*A*
C2—H2*A*⋯O1^i^	0.93	2.42	3.133 (2)	134
C5—H5*A*⋯O1^ii^	0.93	2.38	3.253 (2)	155
C8—H8*A*⋯S1	0.93	2.67	3.1105 (18)	110
C11—H11*A*⋯S2	0.93	2.47	2.9322 (18)	111
C15—H15*A*⋯O2^iii^	0.93	2.58	3.461 (2)	158
C9—H9*A*⋯*Cg*2^iv^	0.93	2.90	3.645 (2)	138

Symmetry codes: (i) 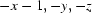; (ii) 
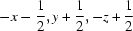
; (iii) 
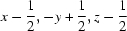
; (iv) 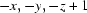. *Cg*2 is the centroid of the N2/C1–C5 ring.

**Fig. 1.**
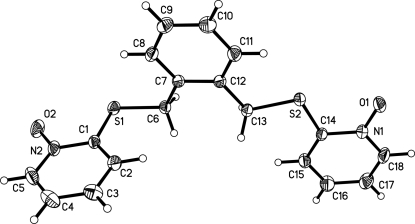
The molecular structure of the title compound with displacement ellipsoids drawn at the 30% probability level for non-hydrogen atoms.

## supplementary crystallographic information

### Comment

Thioether-type ligands are attracting great attention as the conformational freedom, flexible multidentate bridging ligands (Xie *et al.*, 2005). In continuation of the structural study of thioether-type ligands (Han *et al.*, 2005), herein, we report the crystal sructure of the title compound.

The title compound (Fig. 1) was obtained by the reaction of 2-mercaptopyridine N-oxide and *o*-xylylene dibromide. In the asymmetric unit, the central benzene ring makes dihedral angles of 7.44 and 86.52° with the two outer pyridine N-oxygen rings and the crystal packing is stabilized by C—H···O and C—H..S hydrogen bonding, C—H···π interactions (Table 1) and weak π-π staking interactions [centroid-to-centroid distance 3.7596 (7) Å].

### Experimental

2-Mercaptopyridine N-oxide (1.2719 g, 10.00 mmol) was added to a stirred solution of KOH (0.6091 g, 10.85 mmol) in ethanol (50 ml). After 30 min, *o*-xylylene dibromide (1.3206 g, 5.00 mmol) was added and the mixture was heated to 343 K for 6 h with vigorous stirring. The mixture was cooled to room temperature and the precipitate was filtered off and washed with ethanol and water, giving a white powder in 66.0% yield. Then, a solution of the powder in CHCl_3_/CH_3_CN with a molar ratio of 1:1 was filtered. Slow diffusion of ether into the filtrate yielded colourless prism crystals.

### Refinement

The H atoms H6A and H6B of the C6 atom were found from a difference Fourier map and refined freely. The rest H atoms were fixed geometrically with C—H = 0.93-0.97 Å and treated as riding with *U*_iso_(H) = 1.2*U*_eq_(C).

### Crystal data

**Table:**

C_18_H_16_N_2_O_2_S_2_	*F*(000) = 744
*M**_r_* = 356.47	*D*_x_ = 1.470 Mg m^−^^3^
Monoclinic, *P*2_1_/*n*	Mo *K*α radiation, λ = 0.71073 Å
Hall symbol: -P 2yn	Cell parameters from 9866 reflections
*a* = 7.5075 (15) Å	θ = 2.0–27.9°
*b* = 17.810 (4) Å	µ = 0.34 mm^−^^1^
*c* = 12.480 (3) Å	*T* = 293 K
β = 105.20 (3)°	Prism, colourless
*V* = 1610.3 (7) Å^3^	0.34 × 0.28 × 0.16 mm
*Z* = 4	

### Data collection

**Table:**

Bruker SMART CCD area-detector diffractometer	3145 reflections with *I* > 2σ(*I*)
Radiation source: fine-focus sealed tube	*R*_int_ = 0.017
graphite	θ_max_ = 27.9°, θ_min_ = 2.0°
ω scans	*h* = −8→9
9681 measured reflections	*k* = −17→23
3782 independent reflections	*l* = −15→16

### Refinement

**Table:**

Refinement on *F*^2^	Primary atom site location: structure-invariant direct methods
Least-squares matrix: full	Secondary atom site location: difference Fourier map
*R*[*F*^2^ > 2σ(*F*^2^)] = 0.030	Hydrogen site location: inferred from neighbouring sites
*wR*(*F*^2^) = 0.083	H atoms treated by a mixture of independent and constrained refinement
*S* = 1.02	*w* = 1/[σ^2^(*F*_o_^2^) + (0.0371*P*)^2^ + 0.5053*P*] where *P* = (*F*_o_^2^ + 2*F*_c_^2^)/3
3782 reflections	(Δ/σ)_max_ < 0.001
225 parameters	Δρ_max_ = 0.25 e Å^−^^3^
0 restraints	Δρ_min_ = −0.20 e Å^−^^3^

### Special details

**Table:**

Geometry. All e.s.d.'s (except the e.s.d. in the dihedral angle between two l.s. planes) are estimated using the full covariance matrix. The cell e.s.d.'s are taken into account individually in the estimation of e.s.d.'s in distances, angles and torsion angles; correlations between e.s.d.'s in cell parameters are only used when they are defined by crystal symmetry. An approximate (isotropic) treatment of cell e.s.d.'s is used for estimating e.s.d.'s involving l.s. planes.
Refinement. Refinement of *F*^2^ against ALL reflections. The weighted *R*-factor *wR* and goodness of fit *S* are based on *F*^2^, conventional *R*-factors *R* are based on *F*, with *F* set to zero for negative *F*^2^. The threshold expression of *F*^2^ > σ(*F*^2^) is used only for calculating *R*-factors(gt) *etc*. and is not relevant to the choice of reflections for refinement. *R*-factors based on *F*^2^ are statistically about twice as large as those based on *F*, and *R*- factors based on ALL data will be even larger.

### Fractional atomic coordinates and isotropic or equivalent isotropic displacement parameters (Å^2^)

**Table:**

	*x*	*y*	*z*	*U*_iso_*/*U*_eq_	
S1	0.17377 (5)	0.17901 (2)	0.34399 (3)	0.03675 (11)	
S2	−0.30419 (5)	−0.03050 (2)	−0.04963 (3)	0.03289 (10)	
C7	−0.00609 (19)	0.04497 (8)	0.25080 (11)	0.0304 (3)	
N1	−0.47078 (17)	−0.01887 (7)	−0.25627 (10)	0.0350 (3)	
O1	−0.48428 (16)	−0.09127 (6)	−0.24087 (9)	0.0458 (3)	
C14	−0.38096 (18)	0.02425 (8)	−0.16810 (11)	0.0308 (3)	
C6	0.0423 (2)	0.12465 (8)	0.22786 (12)	0.0335 (3)	
C1	0.0008 (2)	0.20710 (8)	0.40531 (11)	0.0324 (3)	
C12	−0.11785 (18)	0.00251 (8)	0.16387 (11)	0.0301 (3)	
C15	−0.3632 (2)	0.10057 (9)	−0.18273 (13)	0.0397 (3)	
H15A	−0.3009	0.1304	−0.1235	0.048*	
N2	0.06622 (19)	0.25129 (7)	0.49662 (10)	0.0402 (3)	
C2	−0.1847 (2)	0.18976 (9)	0.37257 (13)	0.0388 (3)	
H2A	−0.2299	0.1592	0.3111	0.047*	
C11	−0.1519 (2)	−0.07213 (9)	0.18381 (13)	0.0421 (4)	
H11A	−0.2260	−0.1007	0.1270	0.051*	
C13	−0.19734 (19)	0.03928 (8)	0.05228 (11)	0.0318 (3)	
H13A	−0.0999	0.0646	0.0285	0.038*	
H13B	−0.2883	0.0765	0.0587	0.038*	
O2	0.24350 (18)	0.26652 (7)	0.52668 (10)	0.0570 (3)	
C8	0.0643 (2)	0.01115 (9)	0.35310 (12)	0.0402 (3)	
H8A	0.1367	0.0392	0.4112	0.048*	
C9	0.0293 (3)	−0.06342 (10)	0.37091 (14)	0.0500 (4)	
H9A	0.0787	−0.0852	0.4401	0.060*	
C18	−0.5444 (2)	0.01241 (10)	−0.35676 (13)	0.0456 (4)	
H18A	−0.6060	−0.0178	−0.4157	0.055*	
C5	−0.0505 (3)	0.27874 (9)	0.55340 (14)	0.0536 (5)	
H5A	−0.0048	0.3091	0.6151	0.064*	
C3	−0.3033 (3)	0.21749 (10)	0.43040 (16)	0.0511 (4)	
H3A	−0.4284	0.2060	0.4084	0.061*	
C17	−0.5296 (3)	0.08746 (11)	−0.37289 (14)	0.0527 (4)	
H17A	−0.5807	0.1085	−0.4424	0.063*	
C16	−0.4379 (3)	0.13227 (10)	−0.28521 (14)	0.0507 (4)	
H16A	−0.4269	0.1836	−0.2955	0.061*	
C10	−0.0783 (3)	−0.10491 (10)	0.28622 (14)	0.0512 (4)	
H10A	−0.1019	−0.1552	0.2975	0.061*	
C4	−0.2338 (3)	0.26248 (10)	0.52122 (17)	0.0584 (5)	
H4A	−0.3125	0.2819	0.5608	0.070*	
H6A	0.125 (2)	0.1237 (9)	0.1798 (14)	0.042 (4)*	
H6B	−0.062 (2)	0.1528 (10)	0.1903 (14)	0.043 (4)*	

### Atomic displacement parameters (Å^2^)

**Table:**

	*U*^11^	*U*^22^	*U*^33^	*U*^12^	*U*^13^	*U*^23^
S1	0.03174 (19)	0.0384 (2)	0.0364 (2)	−0.00423 (14)	0.00247 (14)	−0.00293 (15)
S2	0.03698 (19)	0.03129 (19)	0.02808 (18)	0.00011 (14)	0.00442 (14)	−0.00330 (13)
C7	0.0319 (7)	0.0324 (7)	0.0271 (7)	0.0032 (5)	0.0081 (5)	−0.0001 (5)
N1	0.0348 (6)	0.0363 (7)	0.0308 (6)	−0.0028 (5)	0.0034 (5)	−0.0047 (5)
O1	0.0547 (7)	0.0324 (6)	0.0443 (6)	−0.0074 (5)	0.0022 (5)	−0.0070 (5)
C14	0.0285 (7)	0.0351 (7)	0.0271 (7)	−0.0009 (5)	0.0044 (5)	−0.0038 (5)
C6	0.0403 (8)	0.0321 (7)	0.0267 (7)	0.0001 (6)	0.0064 (6)	−0.0010 (5)
C1	0.0395 (8)	0.0268 (7)	0.0277 (7)	−0.0026 (6)	0.0033 (6)	−0.0006 (5)
C12	0.0308 (7)	0.0320 (7)	0.0276 (7)	0.0021 (5)	0.0078 (5)	−0.0003 (5)
C15	0.0450 (9)	0.0357 (8)	0.0354 (8)	−0.0046 (6)	0.0054 (6)	−0.0039 (6)
N2	0.0555 (8)	0.0280 (6)	0.0312 (6)	−0.0053 (6)	0.0009 (6)	−0.0017 (5)
C2	0.0385 (8)	0.0371 (8)	0.0390 (8)	−0.0029 (6)	0.0069 (6)	−0.0036 (6)
C11	0.0507 (9)	0.0357 (8)	0.0365 (8)	−0.0049 (7)	0.0053 (7)	0.0004 (6)
C13	0.0333 (7)	0.0323 (7)	0.0271 (7)	−0.0009 (5)	0.0031 (5)	−0.0019 (5)
O2	0.0580 (8)	0.0494 (7)	0.0497 (7)	−0.0150 (6)	−0.0107 (6)	−0.0095 (6)
C8	0.0462 (9)	0.0415 (8)	0.0283 (7)	0.0008 (7)	0.0015 (6)	0.0019 (6)
C9	0.0655 (11)	0.0444 (9)	0.0351 (9)	0.0038 (8)	0.0046 (8)	0.0130 (7)
C18	0.0472 (9)	0.0530 (10)	0.0289 (8)	−0.0032 (8)	−0.0037 (6)	−0.0035 (7)
C5	0.0920 (15)	0.0324 (8)	0.0374 (9)	0.0016 (9)	0.0186 (9)	−0.0065 (7)
C3	0.0484 (10)	0.0446 (9)	0.0644 (12)	0.0024 (8)	0.0223 (9)	0.0019 (8)
C17	0.0617 (11)	0.0541 (11)	0.0348 (9)	−0.0003 (9)	−0.0004 (7)	0.0086 (7)
C16	0.0653 (11)	0.0380 (9)	0.0447 (10)	−0.0039 (8)	0.0071 (8)	0.0055 (7)
C10	0.0695 (12)	0.0348 (8)	0.0467 (10)	−0.0019 (8)	0.0104 (8)	0.0093 (7)
C4	0.0828 (14)	0.0410 (10)	0.0630 (12)	0.0088 (9)	0.0398 (11)	−0.0010 (8)

### Geometric parameters (Å, °)

**Table:**

S1—C1	1.7442 (15)	C2—C3	1.376 (2)
S1—C6	1.8061 (15)	C2—H2A	0.9300
S2—C14	1.7384 (15)	C11—C10	1.382 (2)
S2—C13	1.8084 (14)	C11—H11A	0.9300
C7—C8	1.385 (2)	C13—H13A	0.9700
C7—C12	1.4055 (19)	C13—H13B	0.9700
C7—C6	1.511 (2)	C8—C9	1.383 (2)
N1—O1	1.3116 (16)	C8—H8A	0.9300
N1—C18	1.351 (2)	C9—C10	1.367 (2)
N1—C14	1.3660 (17)	C9—H9A	0.9300
C14—C15	1.383 (2)	C18—C17	1.361 (3)
C6—H6A	0.971 (17)	C18—H18A	0.9300
C6—H6B	0.942 (18)	C5—C4	1.360 (3)
C1—N2	1.3658 (18)	C5—H5A	0.9300
C1—C2	1.380 (2)	C3—C4	1.374 (3)
C12—C11	1.389 (2)	C3—H3A	0.9300
C12—C13	1.5128 (19)	C17—C16	1.383 (2)
C15—C16	1.376 (2)	C17—H17A	0.9300
C15—H15A	0.9300	C16—H16A	0.9300
N2—O2	1.3130 (18)	C10—H10A	0.9300
N2—C5	1.355 (2)	C4—H4A	0.9300
			
C1—S1—C6	101.12 (7)	C12—C11—H11A	119.3
C14—S2—C13	101.52 (7)	C12—C13—S2	110.19 (10)
C8—C7—C12	118.93 (13)	C12—C13—H13A	109.6
C8—C7—C6	122.05 (13)	S2—C13—H13A	109.6
C12—C7—C6	118.93 (12)	C12—C13—H13B	109.6
O1—N1—C18	120.85 (12)	S2—C13—H13B	109.6
O1—N1—C14	118.39 (12)	H13A—C13—H13B	108.1
C18—N1—C14	120.76 (13)	C9—C8—C7	121.54 (14)
N1—C14—C15	119.39 (13)	C9—C8—H8A	119.2
N1—C14—S2	110.69 (10)	C7—C8—H8A	119.2
C15—C14—S2	129.92 (11)	C10—C9—C8	119.57 (15)
C7—C6—S1	117.35 (10)	C10—C9—H9A	120.2
C7—C6—H6A	109.0 (10)	C8—C9—H9A	120.2
S1—C6—H6A	101.6 (10)	N1—C18—C17	120.86 (15)
C7—C6—H6B	112.7 (10)	N1—C18—H18A	119.6
S1—C6—H6B	108.7 (10)	C17—C18—H18A	119.6
H6A—C6—H6B	106.4 (14)	N2—C5—C4	120.81 (16)
N2—C1—C2	119.34 (14)	N2—C5—H5A	119.6
N2—C1—S1	112.67 (11)	C4—C5—H5A	119.6
C2—C1—S1	127.99 (12)	C4—C3—C2	118.96 (17)
C11—C12—C7	118.52 (13)	C4—C3—H3A	120.5
C11—C12—C13	122.19 (13)	C2—C3—H3A	120.5
C7—C12—C13	119.29 (12)	C18—C17—C16	119.52 (16)
C16—C15—C14	119.74 (14)	C18—C17—H17A	120.2
C16—C15—H15A	120.1	C16—C17—H17A	120.2
C14—C15—H15A	120.1	C15—C16—C17	119.72 (16)
O2—N2—C5	121.49 (14)	C15—C16—H16A	120.1
O2—N2—C1	118.23 (13)	C17—C16—H16A	120.1
C5—N2—C1	120.27 (14)	C9—C10—C11	119.92 (15)
C3—C2—C1	120.38 (15)	C9—C10—H10A	120.0
C3—C2—H2A	119.8	C11—C10—H10A	120.0
C1—C2—H2A	119.8	C5—C4—C3	120.23 (17)
C10—C11—C12	121.50 (15)	C5—C4—H4A	119.9
C10—C11—H11A	119.3	C3—C4—H4A	119.9
			
O1—N1—C14—C15	179.24 (13)	S1—C1—C2—C3	−179.40 (13)
C18—N1—C14—C15	−1.0 (2)	C7—C12—C11—C10	−0.2 (2)
O1—N1—C14—S2	−0.70 (16)	C13—C12—C11—C10	180.00 (15)
C18—N1—C14—S2	179.09 (12)	C11—C12—C13—S2	−9.11 (17)
C13—S2—C14—N1	−178.90 (10)	C7—C12—C13—S2	171.08 (10)
C13—S2—C14—C15	1.17 (16)	C14—S2—C13—C12	−179.41 (9)
C8—C7—C6—S1	−7.41 (19)	C12—C7—C8—C9	1.0 (2)
C12—C7—C6—S1	176.13 (10)	C6—C7—C8—C9	−175.47 (15)
C1—S1—C6—C7	−80.37 (12)	C7—C8—C9—C10	−0.5 (3)
C6—S1—C1—N2	−178.97 (10)	O1—N1—C18—C17	−179.67 (16)
C6—S1—C1—C2	1.27 (15)	C14—N1—C18—C17	0.5 (2)
C8—C7—C12—C11	−0.6 (2)	O2—N2—C5—C4	179.98 (16)
C6—C7—C12—C11	175.95 (13)	C1—N2—C5—C4	0.5 (2)
C8—C7—C12—C13	179.20 (13)	C1—C2—C3—C4	−0.1 (3)
C6—C7—C12—C13	−4.23 (19)	N1—C18—C17—C16	0.0 (3)
N1—C14—C15—C16	0.8 (2)	C14—C15—C16—C17	−0.3 (3)
S2—C14—C15—C16	−179.24 (13)	C18—C17—C16—C15	−0.1 (3)
C2—C1—N2—O2	179.46 (13)	C8—C9—C10—C11	−0.3 (3)
S1—C1—N2—O2	−0.33 (17)	C12—C11—C10—C9	0.7 (3)
C2—C1—N2—C5	−1.0 (2)	N2—C5—C4—C3	0.2 (3)
S1—C1—N2—C5	179.20 (12)	C2—C3—C4—C5	−0.4 (3)
N2—C1—C2—C3	0.9 (2)		

### Hydrogen-bond geometry (Å, °)

**Table:**

*D*—H···*A*	*D*—H	H···*A*	*D*···*A*	*D*—H···*A*
C2—H2A···O1^i^	0.93	2.42	3.133 (2)	134
C5—H5A···O1^ii^	0.93	2.38	3.253 (2)	155
C8—H8A···S1	0.93	2.67	3.1105 (18)	110
C11—H11A···S2	0.93	2.47	2.9322 (18)	111
C15—H15A···O2^iii^	0.93	2.58	3.461 (2)	158
C9—H9A···Cg2^iv^	0.93	2.90	3.645 (2)	138

Symmetry codes: (i) −*x*−1, −*y*, −*z*; (ii) −*x*−1/2, *y*+1/2, −*z*+1/2; (iii) *x*−1/2, −*y*+1/2, *z*−1/2; (iv) −*x*, −*y*, −*z*+1.
